# Sublingual Vaccination Induces Mucosal and Systemic Adaptive Immunity for Protection against Lung Tumor Challenge

**DOI:** 10.1371/journal.pone.0090001

**Published:** 2014-03-05

**Authors:** Shailbala Singh, Guojun Yang, Kimberly S. Schluns, Scott M. Anthony, K. Jagannadha Sastry

**Affiliations:** 1 Department of Immunology, The University of Texas M.D. Anderson Cancer Center, Houston, Texas, United States of America; 2 Immunology Graduate Program, The University of Texas Graduate School of Biomedical Sciences at Houston, Houston, Texas, United States of America; Federal University of São Paulo, Brazil

## Abstract

Sublingual route offers a safer and more practical approach for delivering vaccines relative to other systemic and mucosal immunization strategies. Here we present evidence demonstrating protection against ovalbumin expressing B16 (B16-OVA) metastatic melanoma lung tumor formation by sublingual vaccination with the model tumor antigen OVA plus synthetic glycolipid alpha-galactosylceramide (aGalCer) for harnessing the adjuvant potential of natural killer T (NKT) cells, which effectively bridge innate and adaptive arms of the immune system. The protective efficacy of immunization with OVA plus aGalCer was antigen-specific as immunized mice challenged with parental B16 tumors lacking OVA expression were not protected. Multiple sublingual immunizations in the presence, but not in the absence of aGalCer, resulted in repeated activation of NKT cells in the draining lymph nodes, spleens, and lungs of immunized animals concurrent with progressively increasing OVA-specific CD8^+^ T cell responses as well as serum IgG and vaginal IgA levels. Furthermore, sublingual administration of the antigen only in the presence of the aGalCer adjuvant effectively boosted the OVA-specific immune responses. These results support potential clinical utility of sublingual route of vaccination with aGalCer-for prevention of pulmonary metastases.

## Introduction

While radiation, chemotherapy, and surgery are routinely used to manage locally advanced cancers such as melanoma and squamous cell carcinoma of head and neck, the overall success of the treatment is often undermined by the incidence of metastasis at distant locations [1]. Because of the circulatory pattern and the selective affinity of the endothelium for cancer cells, the lung is the second most commonly targeted organ for metastases after liver [2]–[4]. Pulmonary metastases are most frequently observed in cases of melanoma, breast, colorectal, head and neck, prostrate and renal cancers [2]–[4]. Along with the conventional treatment of localized cancer, immunotherapeutic approaches that activate the T cell mediated responses specifically against the tumor can prevent the incidence of pulmonary metastasis [1]. In general, most pre-clinical cancer vaccine studies rely on extrapolating the observations of protective efficacy against subcutaneous tumors to mucosal tumors; however new evidence is emerging on the effectiveness of mucosal immunization to selectively direct the anti-tumor T cells to localize at the sites of mucosal tumors [5], [6].

A large body of experimental evidence, from both rodents and human studies, supports the existence of a common mucosal immune system connecting pulmonary, gastric, and genital mucosal tissues. This affords the possibility of delivering vaccines at one mucosal site that is easy to administer in order to induce immunity in distal mucosal tissues that may be difficult to target [5]–[8]. Among the various mucosal routes for delivery of vaccines being explored, sublingual immunization offers an effective, safer, inexpensive, and non-invasive practical option for vaccination [9]–[11].

In comparison to oro-gastric delivery of antigens, sublingually delivered antigens are absorbed directly into the bloodstream from oral mucosa without gastrointestinal processing, thereby limiting their proteolytic degradation [9]. Furthermore, studies investigating immunotherapies targeting allergies have demonstrated that sublingual route allows safe delivery of antigen without inducing anaphylaxis [12]. Although effective at inducing mucosal immunity, intranasal immunization may promote retrograde transport of antigen and/or adjuvant from vaccine formulations to the brain and other neural tissues, potentially causing side effects such as Bell's palsy, which has been observed in volunteers given an influenza vaccine containing a mutated heat-labile enterotoxin (LT) adjuvant [13]–[16]. This is in contrast to the sublingual route of delivery of influenza vaccine (live or inactivated), wherein no migration or replication of virus to the central nervous system occurred [9], [17].

In the current study, we demonstrate for the first time that in a prophylactic vaccination setting, sublingual immunization is a highly effective strategy for inducing protection against a B16-ovalbumin (B16-OVA) lung tumor challenge in a mouse model. The vaccine formulation included alpha-galactosylceramide (aGalCer), a synthetic glycolipid that selectively and potently activates natural killer T (NKT) cells, which are among the most effective innate immune modulators for inducing activation and maturation of dendritic cells (DC) that in turn induce CD4 and CD8 T cell mediated adaptive immune responses [18]–[23]. Using ovalbumin (OVA) in B16-OVA tumors as a surrogate tumor associated antigen [24], we show that sublingual vaccination with OVA antigen admixed with aGalCer induced persistent antigen-specific T cell responses systemically as well as in the lungs to prevent formation of OVA-expressing B16-melanoma lung tumors.

## Materials and Methods

### Animal Experiments and ethics statement

Female C57Bl/6 mice aged 6–10 weeks were purchased from the National Cancer Institute (Frederick, MD). The animals were maintained in specific pathogen-free environment at the institutional animal facility. The animal facility is fully accredited by the Association for Assessment and Accreditation of Laboratory Animals Care International. All animal procedures were conducted in compliance with the animal care and use protocol (099404437) approved by the Institutional Animal Care and Use Committee (IACUC) at the UT M.D. Anderson Cancer Center, Houston, TX. All manipulations were performed on animals anaesthetized with ketamine (100 mg/kg) and xylazine (10 mg/kg) cocktail administered by i.p. route. The animals were monitored and all efforts were made to minimize suffering. At different times post immunization, the animals were sacrificed according to the institutional guidelines and different organs were collected for immune assays.

### Cell Lines and cell cultures

Murine T lymphoma (thymoma) cell line EL-4 (C57BL/6, H-2b) was maintained in RPMI 1640 (Thermo Scientific Hyclone, Logan, UT), supplemented with 10% heat inactivated FBS (Atlanta Biologicals, Lawrenceville, GA), 50 U/ml of penicillin-streptomycin (Thermo Scientific Hyclone, Logan, UT) and 50 µg/ml gentamycin (Lonza Biowittaker, Walkersville, MD). The B16 melanoma cells expressing ovalbumin (B16-OVA) and parental B16 (B16) melanoma cells were kindly provided by Dr. W. Overwijk (The UT MD Anderson Cancer Center, Houston, TX) and maintained in DMEM (Thermo Scientific Hyclone, Logan, UT), supplemented with 10% FBS and penicillin and streptomycin.

### Reagents

The synthetic peptide corresponding to the H-2^b^-restricted cytotoxic T lymphocyte (CTL) epitope of chicken ovalbumin (SIINFEKL) was purchased from Peptides International Inc. (Louisville, KY), and dissolved in 1× PBS at a concentration of 2.5 mg/ml. The ovalbumin (OVA) protein was purchased from Sigma (St. Louis, MO). The alpha-galactosylceramide (aGalCer) was purchased from Diagnocine LLC (Hackensack, NJ) and dissolved in dimethyl sulfoxide, (Sigma, St. Louis, MO) at a concentration of 1 mg/ml.

### Immunizations

For sublingual immunization, mice were first anesthetized by intraperitoneal (i.p.) injection of ketamine and xylazine hydrochloride [25], [26]. Each animal received an administration of 100 µg of OVA protein either alone or with 2 µg of aGalCer under the tongue using the previously described procedure [10]. To avoid swallowing, the total volume of the inoculum was limited to 7 µl/animal and the animals were maintained with their heads in ante-flexion till they regained consciousness. Mice received three immunizations at 7 day intervals (as depicted in the figures by the vertical arrows pointing downwards) and adaptive immune responses in different tissues were determined at various times post immunization. For evaluation of recall responses, the mice received a booster immunization with either antigen alone or antigen with aGalCer on day 40 after the first immunization and immune responses were determined 7 days later.

### IFN-γ ELISpot Assay

Antigen-specific responses of CD8^+^ T lymphocytes isolated from cervical lymph nodes, lungs, and spleens of the immunized animals at different times post immunization were determined by IFN-γ ELISpot assay as described previously [25], [26]. The cells were stimulated by incubating with either medium alone or ovalbumin peptide (SIINFEKL) (1 µM) or Concavalin A (5 µg/ml) for 48 h before secondary antibody treatment and color development of IFN-γ spot forming cells (SFC) using the commercial reagent kit (BD Biosciences, San Jose, CA). Enumeration of spots representing individual cells producing IFN-γ was done by Zellnet Consulting Inc., Fort Lee NJ using KS-ELISPOT automatic system (Carl Zeiss Inc., Thornwood, NY). Responses were considered positive only when they were above 50 SFC/10^6^ input cells and at least twice the number obtained in cells cultured with medium alone.

### Analyses of antigen specific CTL responses

The antigen-specific CTL response of cells isolated from the spleens of immunized animals was determined by a previously described ^51^Cr Release Assay [25]. Splenocytes were re-stimulated *in vitro* for 5 days with OVA peptide (SIINFEKL) before assaying for cytolytic activity by co-culturing with ^51^Cr-labeled syngeneic EL-4 target cells treated with either OVA peptide or culture medium at different effector: target ratios. The percentage (%) of specific lysis was calculated using the following formula: % specific lysis = (experimental release - spontaneous release)/(maximum release - spontaneous release)×100, where the spontaneous release represents the radioactivity obtained when the target (T) cells were incubated in culture medium without effectors (E) and maximum release represents the radioactivity obtained when the target cells were lysed with 5% Triton X-100.

### Analyses of the phenotype of antigen specific T lymphocytes

Presence of antigen-specific CD8^+^ T cells prior to, and after, boosting immunization was determined using H^2b^ tetramer complexed with the OVA CD8^+^ T cell epitope peptide (SIINFEKL). Briefly, cell were stained with APC-conjugated MHC-I tetramer complexed with OVA peptide (provided by Leo Lefrancois, University of Connecticut), PE-conjugated anti-CD44 (clone IM7 BD Biosciences, San Jose, CA), PerCP Cy5.5 conjugated anti-CD8 (clone 53-6.7 BD Biosciences, San Jose, CA) and FITC-conjugated anti-CD62L (clone MEL-14 BD Biosciences, San Jose, CA) antibodies. Cells were also stained with Aqua Live/Dead reagent (Invitrogen, Carlsbad, CA) to select live cells for all analyses. Percentage of OVA-tetramer positive cells within CD44^hi^ and CD8^+^ live lymphocytes was determined for animals receiving immunization with either OVA alone or OVA+aGalCer.

### Antigen specific antibody response

Antigen specific antibody responses were evaluated in the blood and vaginal washes of immunized animals. Blood samples were collected from the retro-orbital sinuses. Vaginal washes were collected by repeated flushing with PBS. Serum and mucosal secretions were assayed for antibody levels to OVA by ELISA using standard protocols [10]. HRP-conjugated goat antibodies to mouse IgG or IgA (KPL Inc., Gaithersburg, MD) were used for detection. The titer of a sample was defined as the reciprocal of the highest sample dilution yielding an absorbance value at least equal to the sum of the absorbance value of pre-immunization sample plus threefold its standard deviation (SD). For each group of immunized mice, results were expressed as geometric mean titer (GMT) ± SD.

### Analyses of NKT cell and DC activation

Single cell suspensions isolated from the lungs, cervical lymph nodes and spleen of immunized mice were analyzed for activation and proliferation of NKT cells. Cells were stained with Aqua Live/Dead reagent (Invitrogen, Carlsbad, CA), Pacific Blue-conjugated CD3 (clone 500A2, BD Biosciences, San Jose, CA) and the APC-conjugated mouse CD1d tetramer loaded with PBS57 (provided by NIH tetramer facility at Emory University, Atlanta, GA) by the procedure described previously [26]. The activation status of NKT cells isolated from animals at different time points after immunization was determined by intra-cellular staining for IFN-γ production. All the cells were incubated with GolgiPlug reagent (BD Biosciences, San Jose, CA) in complete medium for 4.5 hours prior to cellular staining. Cells were first stained for surface markers and then permeablized for staining with PE-conjugated IFN-γ antibody (BD Biosciences, San Jose, CA) in 1× Perm/Wash Buffer (BD Biosciences, San Jose, CA) [17]. Samples were run on the LSRII flow cytometer and analyses were performed using FlowJo software (Tree Star Inc, Ashland, OR). For NKT cell analysis, lymphocytes were first gated using the forward scatter and side scatter plots. Next live cells were gated using side scatter and Aqua plots. Finally, the NKT cell population was determined by plotting CD3 against the CD1d Tetramer loaded with PBS57 and these CD3^+^ CD1d Tet^+^ cells were further analyzed for cytokine production. For exclusion of cells binding to CD1d tetramer in a non-specific manner, an aliquot of cells from each tissue was stained with APC-conjugated unloaded CD1d tetramer in addition to aqua live/dead reagent and Pacific-blue conjugated CD3.

The activation of DC was analyzed by staining cells isolated from different tissues with FITC-conjugated anti-CD11b (clone M1/70, BD Biosciences, San Jose, CA), APC-conjugated anti-CD11c (clone HL3, BD Biosciences, San Jose, CA) and PE-conjugated anti-CD86 (clone GL1, BD Biosciences, San Jose, CA) antibodies, and incubated for 30 minutes at 4°C. For DC analysis, lymphocytes were first gated using the forward scatter and side scatter plots, followed by determination of median fluorescence intensity (MFI) of CD86 expression on the population of CD11c^+^ cells that included both CD11b^+^ and CD11b^−^ DC [26].

### Protection against challenge with antigen expressing B16 melanoma tumor

To evaluate the efficacy of sublingual immunization with antigen+aGalCer at preventing development of lung tumors animals were immunized prior to inoculation of tumor cells. Four groups of mice (n = 5) were immunized thrice at weekly intervals by sublingual route either with OVA (100 µg/animal) alone, aGalCer (2 µg/animal) alone, OVA (100 µg/animal) admixed with aGalCer (2 µg/animal) or PBS. One week after the last immunization, the animals were challenged with 5×10^4^ B16 melanoma cells expressing OVA (B16-OVA) by the intravenous route. A separate group of animals immunized with OVA admixed with aGalCer was challenged with 5×10^4^ of the parental B16 melanoma cells. Two weeks after tumor challenge, mice were sacrificed and the lung metastases were quantified under a dissecting microscope [27].

### Statistical Analysis

The immune responses and tumor foci were expressed as averages of 3–6 animals/group. Paired two-tailed Student's t- test was used to determine the significance of difference between different immunization groups and correlation between number of immunizations and magnitude of immune response was evaluated by two-way ANOVA. All analyses was performed using GraphPad Prism, version 6 (GraphPad Software, San Diego, CA) and p≤0.05 was considered statistically significant.

## Results

### Multiple rounds of sublingual immunization employing the aGalCer adjuvant induce progressively improving and persistent antigen-specific cell and humoral responses

**Figure 1:**
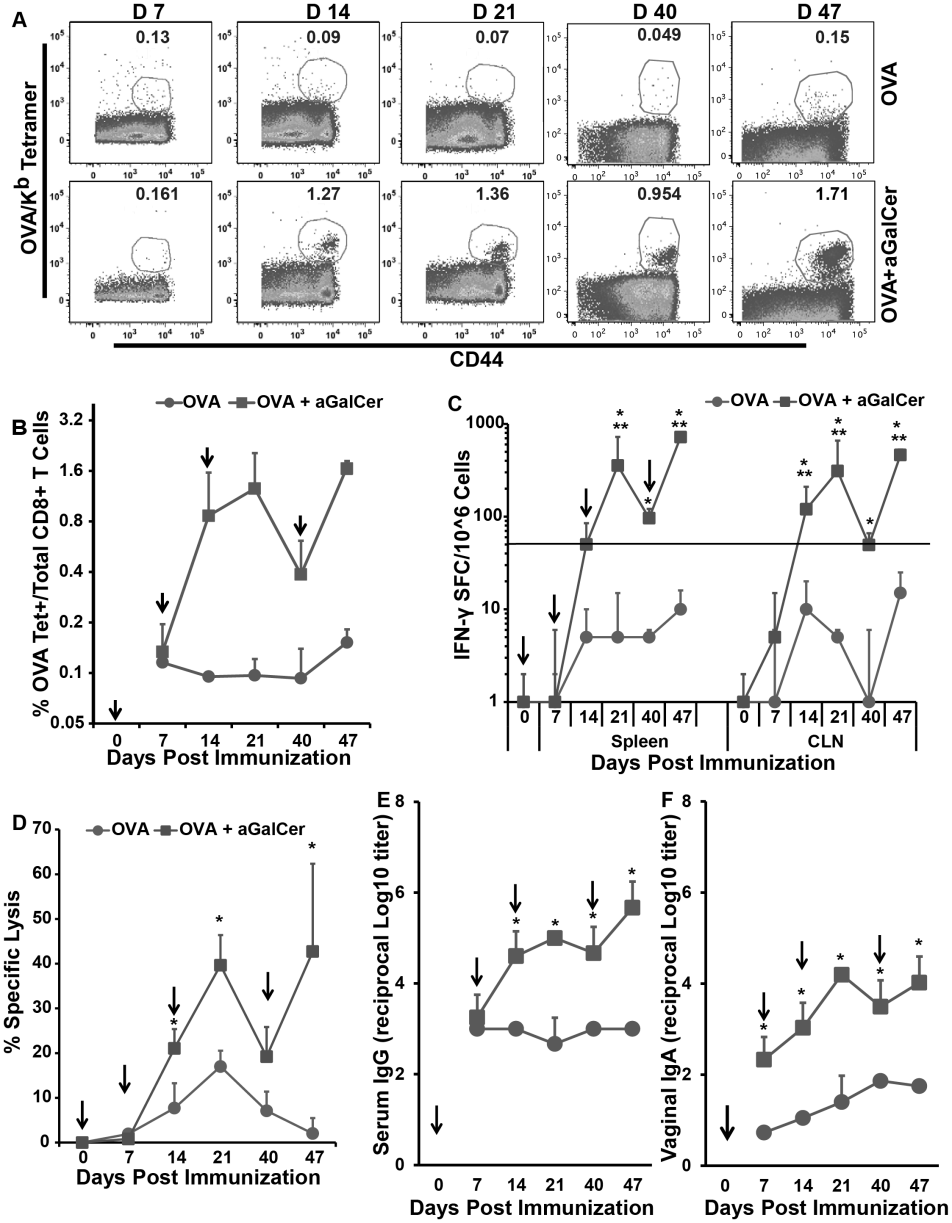
Multiple rounds of sublingual immunization employing the aGalCer adjuvant induce progressively increasing antigen-specific cellular and humoral responses. Effector responses were determined in mice immunized by sublingual route three times at 7 day intervals and boosting on day 41 (i.e. 27 days post last immunization) with OVA or OVA+aGalCer. Vertical arrows at different time points indicate the time of immunization. The kinetics of the development of adaptive immune responses was determined at 7 days post each immunization. (A) Antigen-specific CD8^+^ T lymphocytes were detected in the PBMC by staining with fluorescently labeled OVA/K^b^ tetramer and antibodies to CD44 and CD8, and representative flow plots for OVA/K^b^ tetramer^+^ cells expressed as a percentage of CD8^+^ T lymphocytes from each time point are presented. (B) Cumulative data for percentages of OVA/K^b^ tetramer^+^, CD8^+^ T lymphocytes in PBMC at different time points in mice immunized with OVA or OVA+aGalCer. (C) Single cell suspensions from spleen and CLN were analyzed for antigen-specific IFN-γ production in response to stimulation with the CD8 T cell epitope peptide SIINFEKL from OVA using a standard IFN-γ ELISpot assay. Data are shown as IFN-γ spot forming cells (SFC) per million input cells and OVA-specific responses were adjusted to background medium control and expressed as mean ± S.D. (D) Splenocytes isolated from immunized mice were also analyzed for antigen-specific cytolytic activity by the standard chromium-release assay employing the syngeneic EL-4 target cells pulsed with the OVA peptide, at 100∶1 effector to target cell ratio. Data were adjusted for background by subtracting control values (target cells not pulsed with the OVA peptide) and expressed as mean ±S.D. (E and F) Antigen specific antibody response after each dose was determined by ELISA. The log10 titers of serum IgG and vaginal IgA respectively at different time points in mice immunized with OVA or OVA+ aGalCer were calculated by adjusting to background pre-immune values. Data are expressed as mean ± S.D. and representative of two separate experiments. The statistical significance (p≤0.05), between same number of immunizations with OVA alone and OVA+aGalCer is shown as * and between each additional immunization with either OVA alone or admixed with aGalCer is shown as **.

The effectiveness of sublingual vaccination for the induction of cellular and humoral immune responses was determined by immunizing mice with OVA protein in the presence or absence of aGalCer adjuvant. Animals received three sublingual immunizations at weekly intervals followed by administration of a booster vaccination at day 40 post first immunization. Flow cytometric analyses revealed induction of activated OVA-specific CD8^+^ T cells in the peripheral blood as detected by OVA/K^b^ tetramer^+^ staining and CD44^hi^ expression 14 days post immunization that increased by day 21 (Fig. 1A, B). Additionally, the frequency of OVA-specific CD8 T cells increased further after boosting at day 40 (Fig. 1A, B). Another set of similarly immunized animals were sacrificed one week after each immunization and single cell suspensions from spleens and draining cervical lymph nodes (CLN) were analyzed for antigen specific function. Substantial numbers of IFN-γ producing cells in response to stimulation with the OVA CD8^+^ T cell epitope peptide were observed in both spleen and CLN of mice immunized with OVA+aGalCer that progressively increased with each dose of immunization (Fig. 1C). The effect of the adjuvant was evident as the numbers of IFN-γ^+^ T cells in OVA+aGalCer recipients were significantly higher than those in mice immunized with OVA alone (Fig. 1C). Significantly greater and progressively increasing OVA peptide-specific CTL activity was also observed in the spleens of mice immunized with OVA+aGalCer in comparison to those immunized with OVA alone (Fig. 1D). At the highest effector to target (E∶T) ratio (100∶1), some OVA peptide-specific CTL activity was detected following three immunizations with OVA in the absence of aGalCer, but it was still significantly lower than that observed in mice administered three doses of OVA+aGalCer (Fig. 1D). At all the E∶T ratios tested, the magnitude of average antigen-specific CTL activity was greater for the group of mice immunized three times with OVA+aGalCer compared to mice immunized with OVA alone, but achieved significance only at the 50∶1 ratio. In parallel with the generation of antigen-specific CD8^+^ T cell responses, sublingual immunization with OVA+aGalCer, relative to OVA alone, also resulted in the induction of significantly greater OVA specific serum IgG (Fig. 1E) and vaginal IgA responses (Fig. 1F). Each additional immunization of OVA+aGalCer resulted in an increase in the magnitude of antigen specific IgG and IgA responses.

Since multiple doses of OVA+aGalCer delivered by the sublingual route resulted in progressively increasing OVA-specific immune responses with each dose, we tested whether inclusion or exclusion of aGalCer in subsequent immunization would significantly affect the OVA-specific T cell responses. Linear regression analysis of OVA-specific CD8^+^ T cell responses demonstrated highly statistically significant correlation between the increasing magnitude of the T cell response and the number of immunizations, in the presence but not absence of aGalCer (Fig. S1). Together, these results strongly support the effectiveness of sublingual immunization for the induction of adaptive immune responses as well as underscore the importance of including aGalCer during boosting.

### Activation of NKT cells repeatedly with each dose of aGalCer delivered by the sublingual route

**Figure 2:**
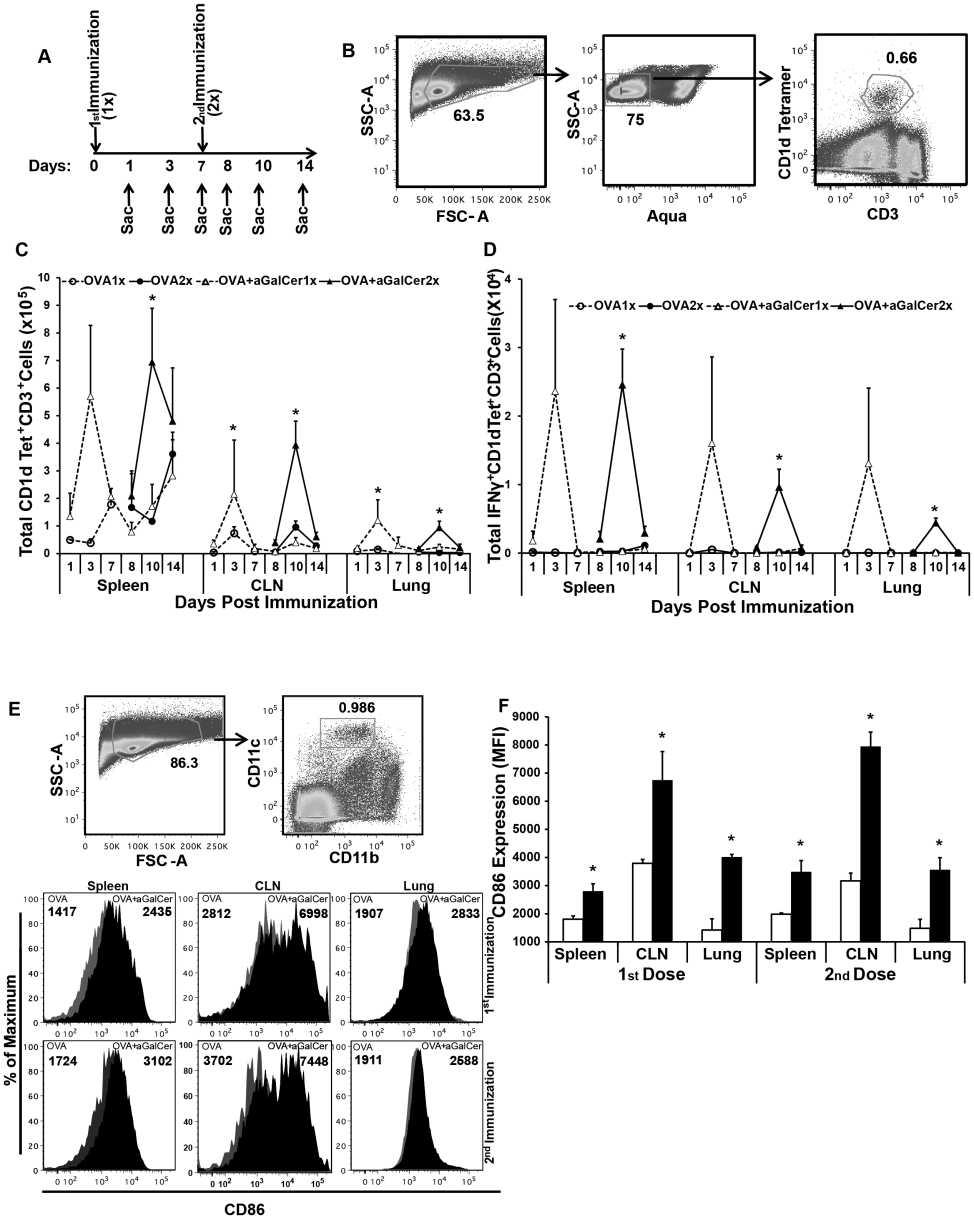
Repeated activation of NKT and dendritic cells with each immunization employing the aGalCer adjuvant delivered by the sublingual route. (A) Mice were immunized by sublingual route with one or two doses of OVA or OVA+aGalCer at 7 day intervals and sacrificed at different time points as shown to determine activation of NKT cells and DC. (B) Gating strategy for staining NKT cells isolated from the spleens, CLNs and lungs with fluorescently labeled NKT tetramer, antibodies to CD3 and IFN-γ, and Aqua live/dead stain. (C) The total number of NKT cells (CD1d tetramer^+^ CD3^+^) at different times post immunization in each tissue. (D) The total number of activated of NKT cells in each tissue were determined at different times post immunization by intracellular staining for IFN-γ. (E) Gating tree and representative histograms for CD86 expression on CD11c^+^ cells (activated DC) from mice immunized with OVA+aGalCer (black) in comparison to that from mice immunized with OVA alone (gray) after one or two immunizations (1× and 2×, respectively) are shown. (F) Cumulative data for activated DC from spleens, CLNs and lungs of mice immunized with OVA+aGalCer (black) compared to animals immunized with OVA alone (white) was evaluated by measuring the MFI of CD86 expression on CD11c^+^ cells at day 3 after either 1^st^ or 2^nd^ immunization (i.e. day 3 and day 10 respectively). Data are representative of two separate experiments and expressed as mean ± S.D. The statistical significance (p≤0.05) between groups of mice that were immunized with OVA alone and OVA+aGalCer after 1^st^ and 2^nd^ immunization at different time points is shown as *.

Since aGalCer specifically and potently activates NKT cells [21], [28]–[30] and its inclusion was important for the induction of antigen-specific immune responses in subsequent sublingual immunization (Fig. 1), we next tested whether NKT cells were reactivated during the secondary immunization. Mice were immunized by the sublingual route one or two times at 7-day interval with OVA alone or OVA+aGalCer and sacrificed at 1, 3, and 7 days after delivery of each dose (Fig. 2A). Cells were isolated from spleen, CLN and lung tissues and analyzed for NKT cell expansion and NKT cells specific IFN-γ production. Total NKT cells were enumerated by flow cytometric staining of CD1d tetramer^+^ CD3^+^ T cells (Fig. 2B). In all three tissues analyzed, the frequency of NKT cells was increased at day 3 after each sublingual dose of OVA+aGalCer relative to OVA alone (Fig. 2C). The expansion in the population of NKT cells was significant in CLN and lung with aGalCer administration. The level of expansion and IFN-γ production by NKT cells did not differ within each tissue between the first and the second sublingual immunizations (Fig. 2D). Since the adjuvant role of NKT cells in modulating adaptive immune response relies on the activation of APC, we also determined the effect of aGalCer co-administration on the activation of DC isolated from spleens, CLN and lungs by evaluating CD86 expression on CD11c^+^ cells (Fig. 2E). Concurrent with the activation of NKT cells after each sublingual immunization with aGalCer, we also observed significant enhancement of CD86^+^expression on CD11c^+^ DC in all the three tissues in mice immunized with OVA+aGalCer, relative to OVA alone (Fig. 2F). The MFI of CD86 expression of DC isolated from OVA immunized mice was not significantly different from that of naïve mice. Altogether, these results show that multiple sublingual immunizations employing the aGalCer adjuvant repeatedly activate NKT cells and DC resulting in progressively enhancing antigen-specific cellular and humoral immune responses.

### Anti-tumor efficacy of sublingual vaccination employing the aGalCer adjuvant

**Figure 3:**
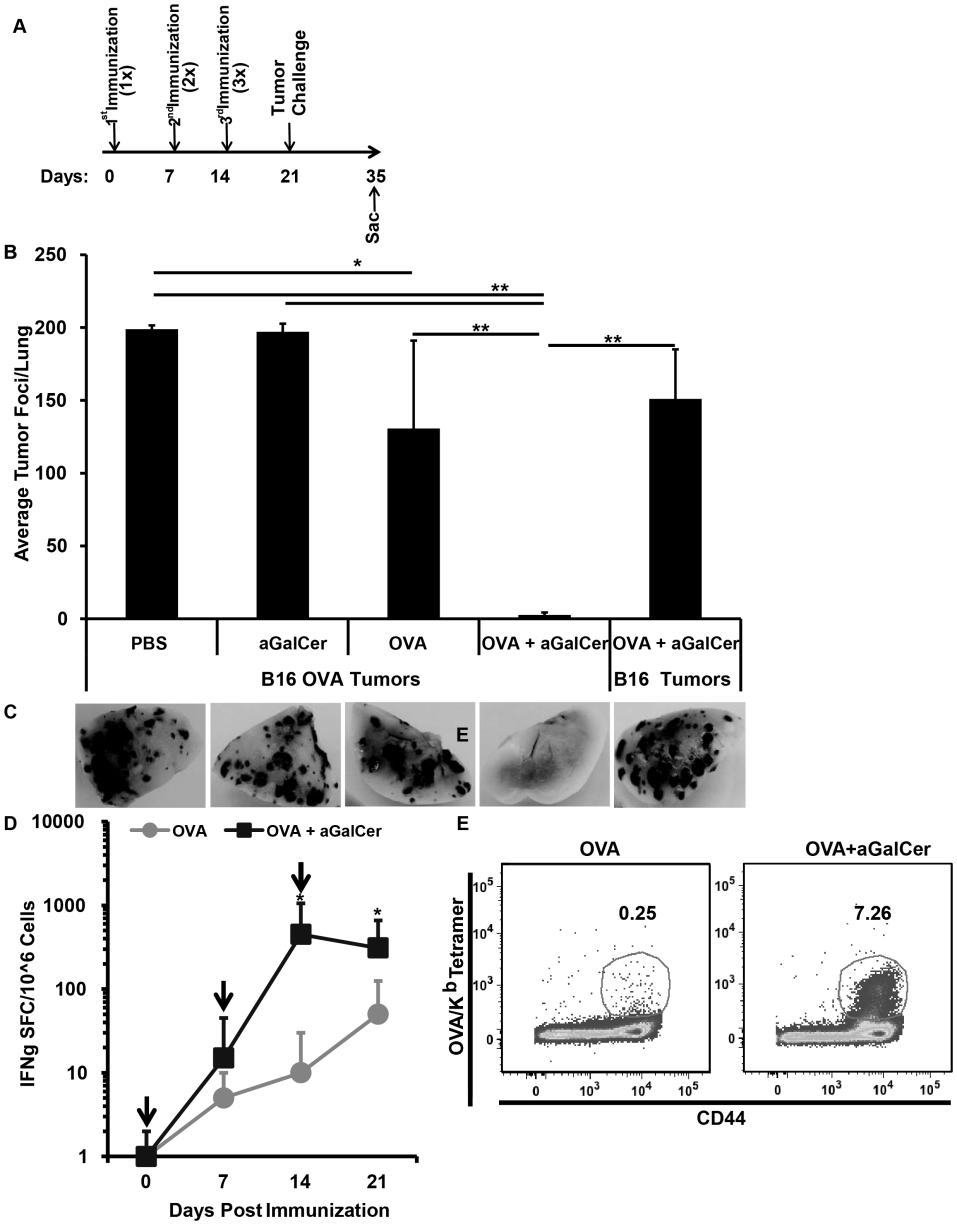
Efficacy of antigen-specific immune responses induced by sublingual immunization employing the aGalCer adjuvant against lung tumor challenge. (A) Mice were immunized three times by sublingual route with either OVA admixed with aGalCer, OVA alone, aGalCer alone or PBS on days 0, 7 and 14. Seven days after final immunization, the mice were challenged by the intravenous route with 5×10^4^ control or OVA-transgenic B16 tumor cells (B16 and B16-OVA, respectively) and lungs were harvested 14 days post challenge to determine the number of tumor foci. (B) Numbers of tumor foci/lung were shown as mean ± S.D. for each of the different groups of mice. Statistical analyses between different groups were performed using student t-test between different treatments and the different levels of significance are shown as * (p≤0.05) and ** (p≤0.001). (C) Representative lungs corresponding to the different groups of mice in panel B. (D) In a separate group of similarly immunized mice, single cell suspensions from the lungs were analyzed 7 days post each immunization including at the time of tumor challenge (day 21) for antigen-specific IFN-γ production in response to stimulation with the CD8 T cell epitope peptide SIINFEKL from OVA using a standard IFN-γ ELISpot assay. Vertical arrows represent the time of immunization and data are shown as IFN-γ spot forming cells (SFC) per million input cells and OVA specific responses were adjusted to background medium control and expressed as mean ± S.D. The statistical significance (p≤0.05) between groups of mice that were immunized with OVA alone and OVA+aGalCer at different time points is shown as *. (E) Representative dot plots showing antigen specific effector CD8 T lymphocyte population (OVA/K^b^ tetramer^+^, CD44^hi^ cells) in the lungs at the time of tumor challenge (day 21) for mice immunized with OVA alone and OVA admixed with aGalCer.

While sublingual vaccinations successfully induced protective immunity against respiratory infection with influenza virus [10], [11], [17], [31], the efficacy of sublingual immunization in eliminating lung tumors has not been demonstrated. To assess the protective efficacy of adaptive immunity induced by sublingual immunization employing the aGalCer adjuvant, as demonstrated in our studies (Figs. 1 and 2), we employed the B16 melanoma tumor model; specifically B16 tumor cells expressing OVA (B16-OVA) as a surrogate for tumor associated antigen. Separate groups of mice (n = 5) received three immunizations of PBS, OVA alone, aGalCer alone, or OVA+aGalCer at 7 day intervals followed by intravenous challenge with either B16 or B16-OVA tumor cells (5×10^4^ cells) 7 days after the last immunization. The effectiveness of different treatments was determined by enumerating the number of tumor foci formed on the lungs 2 weeks post-challenge (Fig. 3A). Significantly lower average numbers (0–4 tumor foci/animal) of B16-OVA tumors were observed on the lungs of animals immunized with OVA+aGalCer as compared to animals receiving PBS, OVA alone, or aGalCer alone (Figs. 3B and 3C). The protective efficacy of immunization with OVA+aGalCer was antigen-specific because immunized animals challenged with the parental B16 tumors that do not express OVA showed significantly greater numbers of tumor foci (102–178 tumor foci/lung) compared to those challenged with B16-OVA tumors (0–4 tumor foci/lung). Although immunization with OVA alone also resulted in a significant reduction in the average number of tumor foci (81–200 tumor foci/lung) compared to control groups of mice immunized with either PBS or aGalCer, the number of tumor foci were still significantly greater than those in mice immunized with OVA+aGalCer (Fig. 3B). In mice immunized with OVA+aGalCer, majority of animals did not have any tumors. Corresponding to the virtual absence of tumor foci, we observed higher numbers of IFN-γ spot forming units (Fig. 3D) and OVA/K^b^ tetramer^+^ T lymphocytes (Fig. 3E) in the lungs at the time of tumor challenge (day 21) in mice immunized with OVA+aGalCer, relative to those immunized with OVA alone.

## Discussion

Overall, this investigation has demonstrated the effectiveness of sublingual route of vaccination using the aGalCer adjuvant for inducing strong adaptive immunity that afforded significant protection against tumor formation in the lungs. Targeting vaccine-mediated protective immunity to lungs is highly desirable because lungs are a site of primary tumor formation as well as the second most common organ site for the tumor metastasis [1]–[4]. Our studies provide the first evidence of the effectiveness of sublingual route of immunization for lung tumor protection lending support for its application towards protection against tumor metastases that target the lung tissue.

Data from this investigation also emphasizes the importance of including the aGalCer adjuvant in the immunization regimen. Although the adjuvant effect of aGalCer by systemic immunization is known to be highly effective, anergy of NKT cells is induced after only a single administration by systemic route, thus limiting the utility and efficacy of using aGalCer for repeated application [32], [33]. In contrast to this, we show here that multiple doses of the OVA+aGalCer mixture delivered by the sublingual route induced repeated activation of NKT cells and DC along with the induction of strong antigen-specific systemic and mucosal antibody and T cell responses. These results are consistent with and extend our earlier reports showing mucosal intranasal delivery of aGalCer, as opposed to systemic intravenous route, to repeatedly activate NKT cells and prime efficient adaptive immune responses to co-administered antigens [25], [26]. Furthermore, we observed that after sublingual immunization employing the aGalCer adjuvant the activation and expansion of NKT cells was not accompanied by the down regulation in the expression of T cell receptor (TCR) of NKT cells. This is similar to our earlier study employing aGalCer adjuvant for intranasal immunization but in stark contrast to systemic route of aGalCer administration where we and others reported a decrease in the NKT cell population after one day coinciding with down regulation of TCR on NKT cells [26], [34]. Similar kinetics of activation of NKT cells without TCR down regulation in the draining cervical lymph nodes was also observed previously by Kamijuku et al with intranasal administration of aGalCer [34].

Even though strong immune responses can be induced after immunization by the intranasal as well as sublingual route, literature reports point to the associated potential risks of retrograde passage of antigen to central nervous system after intranasal but not sublingual vaccination [9]–[11]. Therefore, sublingual immunization with the advantages of being non-invasive and convenient represents desirable and promising strategy for administration of tumor vaccines for the prevention of pulmonary metastases.

In addition to the stimulation of effector T cell responses, the inclusion of aGalCer as an adjuvant also generated antigen specific CD44^hi^ CD8^+^ T cells that could be re-stimulated with a booster administration of antigen with aGalCer resulting in a further expansion of antigen-specific CD8 T cells. These results are consistent with previously published reports demonstrating the generation of memory CTL responses against influenza virus and cytomegalovirus when aGalCer was included in the vaccine administered by systemic subcutaneous and intraperitoneal routes, respectively [35], [36]. Those studies established the correlation of increased expression of the pro-survival gene bcl-2 with the presence of improved memory T cell responses in the presence of aGalCer [35].

Immunohistochemical mapping has revealed that murine lingual immune system is devoid of any organized lymphoid structure and lacks any detectable NKT cells, B cells or CD8^+^T cells [37]–[39]. The sublingual mucosa contains only MHC class II^+^ macrophages and DC subsets that serve as antigen presenting cells [11], [37]–[39]. These observations along with the knowledge that the induction of anergy in NKT cells is a function of aGalCer presentation mediated by B cells and not by DC [32] may explain the ability of NKT cells in different tissues to be reactivated following repeated aGalCer administration by sublingual route of immunization.

In the sublingual mucosa, the primary antigen presenting cells are CCR7^+^ DC that respond and migrate towards CCL19 and CCL21 producing epithelial tissue followed by draining to the cervical lymph nodes to activate lymphocytes, which thereafter migrate to CCL28 expressing tissues such as respiratory tract and genital tract [37], [40]–[42]. In accordance with these reports, our results show the presence of activated CD11c^+^ DC in CLN, lungs and spleens 3days after sublingual delivery of antigen along with aGalCer followed by induction of antigen specific effector responses in these tissues.

In summary, the sublingual route of immunization employing the aGalCer adjuvant provides not only a safer and easier vaccination approach but also enables harnessing the potential of NKT cells to significantly improve antigen-specific immunity, specifically targeting the lung tissue for protection against primary and/or metastatic tumors.

## Supporting Information

Figure S1 **Induction of progressively improving antigen-specific immune responses requires inclusion of aGalCer adjuvant.** Mice were immunized three times by sublingual route with either OVA alone (OVA/OVA/OVA) or with OVA+aGalCer (OVA+aGalCer/OVA+aGalCer/OVA+aGalCer) or with OVA alone twice followed by third immunization using OVA+aGalCer (OVA/OVA/OVA+aGalCer) or with OVA+aGalCer twice followed by third immunization with OVA only (OVA+aGalCer/OVA+aGalCer/OVA). Antigen specific immune responses in the spleen and CLN were evaluated 7 days after the third immunization using mouse IFN-γ ELISPOT assay and linear regression analyses. Data are representative of two separate experiments with 3 mice in each group. The statistical significance (p≤0.05) between groups of mice immunized with additional doses of OVA+aGalCer are shown with asterisks (*).
(TIF)
