# Supplementary figures and images for: Sublingual Vaccination Induces Mucosal and Systemic Adaptive Immunity for Protection against Lung Tumor Challenge
Source: PLoS One. 2014 Mar 5;9(3):e90001. doi: 10.1371/journal.pone.0090001 (PMC3943861; doi:10.1371/journal.pone.0090001)

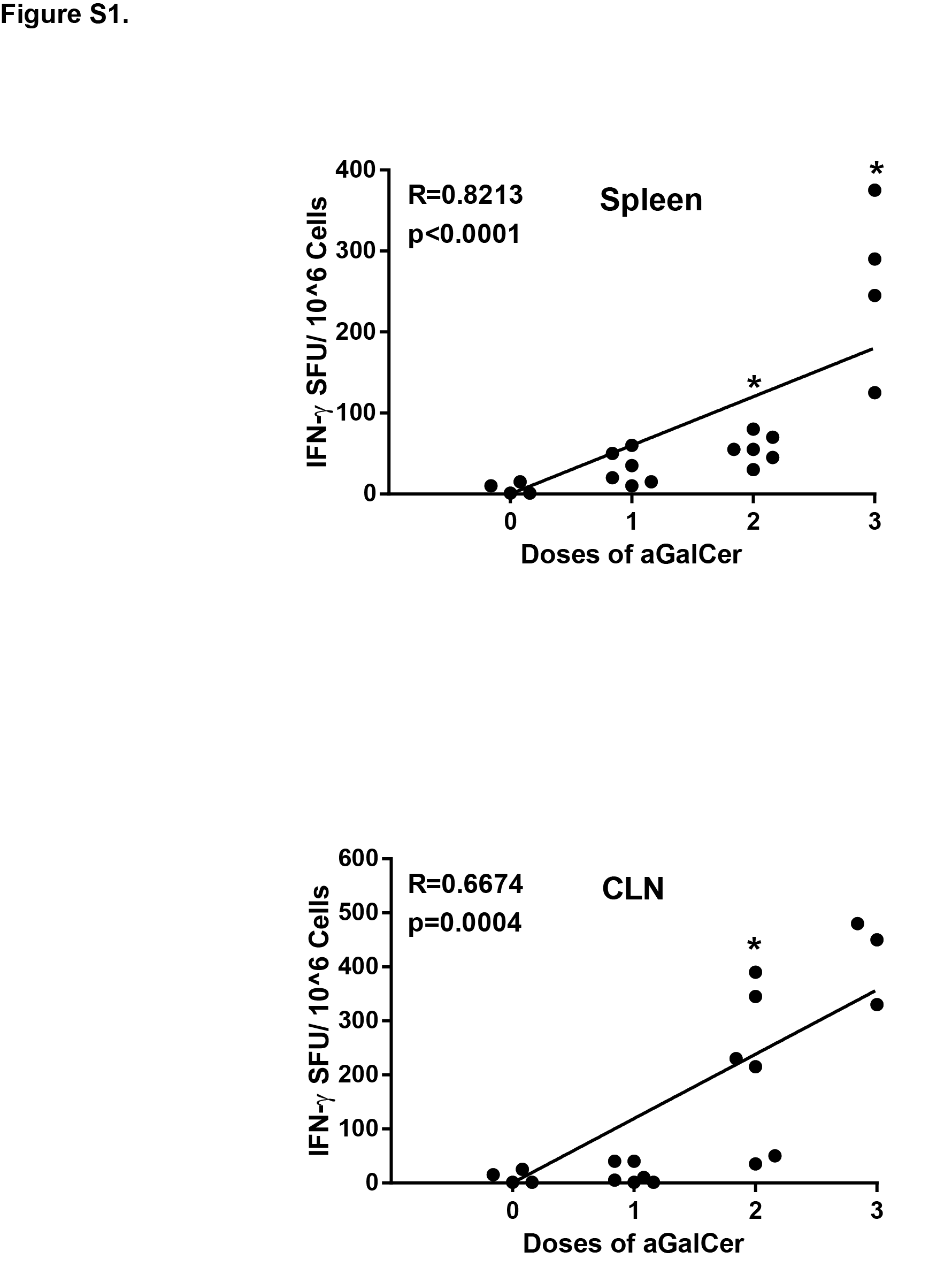

Supplement: Figure S1 — Induction of progressively improving antigen-specific immune responses requires inclusion of aGalCer adjuvant. Mice were immunized three times by sublingual route with either OVA alone (OVA/OVA/OVA) or with OVA+aGalCer (OVA+aGalCer/OVA+aGalCer/OVA+aGalCer) or with OVA alone twice followed by third immunization using OVA+aGalCer (OVA/OVA/OVA+aGalCer) or with OVA+aGalCer twice followed by third immunization with OVA only (OVA+aGalCer/OVA+aGalCer/OVA). Antigen specific immune responses in the spleen and CLN were evaluated 7 days after the third immunization using mouse IFN-γ ELISPOT assay and linear regression analyses. Data are representative of two separate experiments with 3 mice in each group. The statistical significance (p≤0.05) between groups of mice immunized with additional doses of OVA+aGalCer are shown with asterisks (*). (TIF) [file pone.0090001.s001.tif]
